# Fast Contour-Tracing Algorithm Based on a Pixel-Following Method for Image Sensors

**DOI:** 10.3390/s16030353

**Published:** 2016-03-09

**Authors:** Jonghoon Seo, Seungho Chae, Jinwook Shim, Dongchul Kim, Cheolho Cheong, Tack-Don Han

**Affiliations:** 1 Software Platform R&D Lab., LG Electronics Advanced Research Institute, 19 Yangjae-daero 11-gil, Seocho-gu, Seoul, 06772, Korea; jonghoon.seo@lge.com; 2 Department of Computer Science, Yonsei University, 50 Yonsei-ro Seodaemun-gu, Seoul, 03722, Korea; seungho.chae@msl.yonsei.ac.kr (S.C.); jin99foryou@msl.yonsei.ac.kr (J.S.); dckim@msl.yonsei.ac.kr (D.K.); balgeum00@msl.yonsei.ac.kr (C.C.)

**Keywords:** contour tracing, boundary following, pixel following, contour data compression

## Abstract

Contour pixels distinguish objects from the background. Tracing and extracting contour pixels are widely used for smart/wearable image sensor devices, because these are simple and useful for detecting objects. In this paper, we present a novel contour-tracing algorithm for fast and accurate contour following. The proposed algorithm classifies the type of contour pixel, based on its local pattern. Then, it traces the next contour using the previous pixel’s type. Therefore, it can classify the type of contour pixels as a straight line, inner corner, outer corner and inner-outer corner, and it can extract pixels of a specific contour type. Moreover, it can trace contour pixels rapidly because it can determine the local minimal path using the contour case. In addition, the proposed algorithm is capable of the compressing data of contour pixels using the representative points and inner-outer corner points, and it can accurately restore the contour image from the data. To compare the performance of the proposed algorithm to that of conventional techniques, we measure their processing time and accuracy. In the experimental results, the proposed algorithm shows better performance compared to the others. Furthermore, it can provide the compressed data of contour pixels and restore them accurately, including the inner-outer corner, which cannot be restored using conventional algorithms.

## 1. Introduction

A contour is defined as a segment that is one pixel wide and one or more pixels in length, and a boundary is defined as an unbroken contour [1]. Contours and boundaries provide very important information for object representation and image recognition. For example, they are used to separate objects from their backgrounds, to calculate the sizes of objects, to classify shapes and to find the feature points of objects using the length and shape of their contour pixels [2,3]. Moreover, in the field of graphics and vision, it is possible to use the contour information to save the shape of objects and restore them to their original shapes for various applications. Therefore, there have been many studies on contour-tracing algorithms for extracting and tracing the contour of an object. Most of the algorithms are binary image-based contour-tracing algorithms [3,4,5,6,7,8,9], which trace contours on digitized black and white images taken from various image sensors.

In recent years, with the increasing popularity of smart/wearable mobile sensor devices [10], such as smart phones, smart watches and smart glasses, various real-time applications, such as image code recognition, face recognition, optical character recognition (OCR), logo recognition, augmented reality (AR) and mixed reality (MR), have emerged for sensor computing [11,12,13,14,15]. Because smart/wearable mobile sensor devices possess limited hardware resources, such as low-performance processors, small-sized memory, low-resolution displays and low battery capacity, they require simple and fast algorithms for image processing.

Generally, a contour tracing algorithm can be evaluated based on the following four criteria: (1) the accuracy of contour tracing; (2) processing time; (3) data size to save the traced contour information; and (4) the ability to accurately restore and enlarge the original contour using the saved data. However, few studies on contour-tracing algorithms have sought to satisfy all of these criteria. Some of the conventional algorithms miss contour pixels that are at specific relative pixel locations, and others require considerable processing time to trace the pixels, because shortcuts to the local patterns are not considered [7,16]. Moreover, most of the algorithms have no data-compression capabilities that enable them to save the contour information, and some of them cannot restore the contour perfectly using the saved data [17].

In this paper, we propose a novel contour-tracing algorithm based on pixel following that overcomes the above-mentioned problems, *i.e.*,: (1) it provides fast and accurate results for contour-pixel tracing; (2) contour information can be compressed to reduce the memory size; and (3) it accurately restores the compressed data to the original contour image. In order to achieve the objectives, the proposed algorithm initially distinguishes the local patterns made by adjacent contour pixels, and it then finds the next contour pixel that will be traced from the pattern.

The paper is organized as follows. In Section 2, we categorize conventional contour-tracing algorithms and introduce their characteristics. Then, in Section 3, we analyze their performance based on the accuracy and speed. Subsequently, in Section 4, the proposed algorithm is described, and its contour tracing procedure, contour data compression technique and restoration technique are presented. In Section 5, we present a comparison of the conventional algorithms and the proposed algorithm on the basis of their performance, along with experimental results that include the number of traced pixels and the processing times for real-time large-sized images. Moreover, we compare the compressed data size and its restored results with the original traced contour pixels. Finally, in Section 6, we summarize the characteristics and experimental results of the proposed algorithm.

## 2. Related Works

To determine whether a pixel has a dark or light intensity, most conventional contour-tracing algorithms process using 2D binary images that consist of black and white pixels through binarization. In the digitized image, we can assume that the objects have black pixels and that the background has white pixels; therefore, a contour pixel is black, and it has at least one adjacent pixel that is white.

### 2.1. Overview

**Figure 1:**
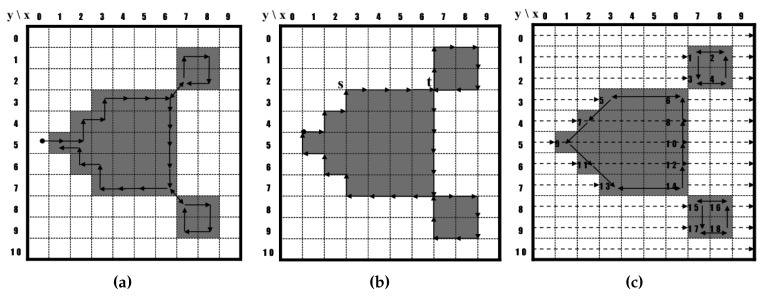
Category of contour tracing algorithms. (**a**) Pixel-following algorithm; (**b**) vertex-following algorithm; (**c**) run-data-based following algorithm.

Figure 1 shows examples of contour traces that were obtained using the contour-tracing algorithms. The conventional contour algorithms can typically be categorized into three types as follows: pixel following, vertex following and run-data-based following [17,18,19]. Of these, the pixel-following method is the most common.

#### 2.1.1. Pixel-Following Method

The pixel-following method traces contour pixels in a predefined manner and then saves their coordinates in memory according to the trace order. In Figure 1a, the algorithm traces contour pixels along the clockwise direction from the current pixel, *i.e.*, it sequentially searches adjacent black pixels of the current pixel using a relative directional order, such as left, front-left, front, front-right, right, rear-right and rear. Pixel-following methods, such as the simple boundary follower (SBF) [4,5,6], modified SBF (MSBF) [3], improved SBF (ISBF) [7], Moore-neighbor tracing (MNT) [20], the radial sweep algorithm (RSA) [9] and the Theo Pavlidis algorithm (TPA) [8], have simple rules for tracing contour pixels based on a chain code. These methods require a frame-size memory to trace the contour and generate erroneous images when the contour image is enlarged [17] because they maintain only the pixel coordinates.

#### 2.1.2. Vertex-Following Method

The vertex-following method traces the vertices of the contour pixels that are located on the edges between the contour pixels and the background pixels [17]. Its procedure is similar to that of the pixel-following method because it uses a chain code and requires a frame-size memory for contour tracing; however, it traces the corners of the contour pixels and their connected edges. Moreover, it stores the corner points of the contour pixels in order to save the traced contour information, and the data can be compressed by reducing the number of points in a straight line. For example, in Figure 1b, five points of the contour from s to t can be stored as there are only two corner points, *i.e.*, (2.5,2.5) and (6.5,2.5) based on the (x,y) coordinate system. Moreover, when the contour images are enlarged, the vertex-following method can provide the correct image [17] because the traced points form the boundaries of the contour.

#### 2.1.3. Run-Data-Based Following Method

The run-data-based following method, which involves the edge-point tracing of run data [17,19], uses run data in pairs consisting of an object’s left and right edges, which are obtained using horizontal scan lines from left to right on an image. The object can have an outer contour and additional inner contours. Therefore, there are five types of run data: (left edge of outer contour, right edge of outer contour), (left edge of outer contour, left edge of inner contour), (right edge of inner contour, left edge of inner contour), (right edge of inner contour, right edge of outer contour) and (right edge of outer contour, left edge of outer contour). For contour following, the run-data-based following method constructs a run-following relationship between the edge points of two adjacent scan lines. In Figure 1c, Scan Line #3 detects (left edge of 5, right edge of 6), and Scan Line #4 detects (left edge of 7, right edge of 8). Subsequently, the run-following relationships between 5 and 7 and between 8 and 6 are generated. The method uses only one or two line buffers and therefore requires a smaller amount of memory compared to the pixel-following and vertex-following methods. Examples of this method are the run-type direction code (RD code) method [17], the PXY table-based method [19] and the OpenCV method [21].

Table 1 lists the characteristics of the contour-following methods. The pixel-following method and vertex-following method trace contours without scanning all of the pixels of the image, and their transition data, such as contour points and the tracing sequence, are generated automatically by the contour-following process. Therefore, only a few pixels need to be scanned in order to obtain the first contour pixel representing the starting point of the object. Despite these merits, they are not suitable for large images with many objects because they require more operations and a larger memory when compared to the run-data-based following method. In other words, they scan all of the pixels with an image-buffer size memory in order to obtain all of the objects, and they have several additional operations that enable them to detect whether to follow a contour pixel for all of the contour pixels and their adjacent background pixels.

On the contrary, the run-data-based following method searches the edge points with a smaller memory and constructs run-following relationships between edge points. Therefore, the traced run-following results are changed iteratively while the edge points are updated. This method is not simple, but it is faster than the other methods for large-scale images, because it scans all of the pixels once; and it does not require any additional operations to be carried out on the pixels. Hence, it is suitable for large-scale image-based applications involving a large number of objects, such as document recognition [17].

### 2.2. Conventional Contour Tracing Algorithms

Let *I* be a binary digital image with M×N pixels, where the coordinate of the top-leftmost pixel is (0,0) and that of the bottom-rightmost pixel is (M−1,N−1). In *I*, a pixel can be represented as P=(x,y),x=0,1,2,⋯,M−1,y=0,1,2,⋯,N−1. Most contour-tracing algorithms use a tracer T(P,d) with absolute directional information d∈{N,NE,NW,W,SW,S,SE,E,NE}, and they have the following basic sequence:

1. The tracer starts contour tracing at the contour of an object after it saves the starting point along with its initial direction.
2. The tracer determines the next contour point using its specific rule of following paths according to the adjacent pixels and then moves to the contour point and changes its absolute direction.
3. If the tracer reaches the start point, then the trace procedure is terminated.

**Figure 2:**
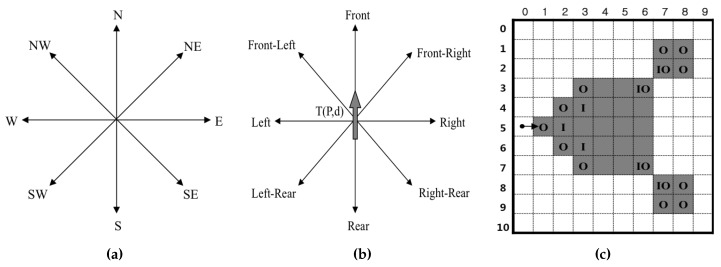
Directions and types of contour pixels. (**a**) Absolute direction *d*; (**b**) relative direction *r*; (**c**) types of contour pixels: inner corner pixel (*I*), outer corner pixel (*O*) and inner-outer corner pixel (IO).

To determine the next contour point, which may be a contour pixel or pixel corner, the tracer detects the intensity of its adjacent pixel $P_{r}$ and the new absolute direction $d_{r}$ for $P_{r}$ by using relative direction information r∈{front,front−left,left,rear−left,rear,rear−right,right, r∈{front−right}. For example, if the absolute direction of the current tracer T(P,d) is *N*, the left direction of the tracer $d_{Left}$ is *W*. Similarly, the left pixel of tracer $P_{Left}$ is (x−1,y). Figure 2a,b show the directional information of the tracer, and Figure 2c shows the different types of contour pixels. The contour pixels can be classified into four types, namely straight line, inner corner pixel, outer corner pixel and inner-outer corner pixel. In Figure 2c, “*O*” represents the outer corner, “*I*” represents the inner corner and “IO” represents the inner-outer corner according to the local pattern of the contour.

In this study, we focus on a contour-tracing algorithm that is suitable for cases involving a relatively small number of objects and that require real-time tracing, such as augmented reality (AR), mixed reality (MR) and recognition image-based code in small-scale images, e.g., a mobile computing environment. Hence, we first introduce and briefly describe the conventional contour-tracing algorithms that are used in this environment and analyze their tracing accuracy and characteristics.

#### 2.2.1. Simple Boundary Follower

The simple boundary follower (SBF) is also known as Papert’s turtle algorithm [6] and as a square-tracing algorithm [22], and it is the simplest contour-tracing algorithm. Initially, the location of tracer *S* is saved, and the tracer moves in a left or right direction. If the pixel tracer is located on a contour pixel, the tracer moves left; otherwise, it moves right. The procedure is shown in Algorithm 1.

**Algorithm 1** Algorithm of the simple boundary follower.1:**procedure** SBF2:    T(P,d)←S(P,d)3:    **do**4:        **if** P = black **then**5:           $T\left(P,d\right)\leftarrow T\left(P_{Left},d_{Left}\right)$6:        **else**7:           $T\left(P,d\right)\leftarrow T\left(P_{Right},d_{Right}\right)$8:    **while** T(P,d)≠S(P,d)

#### 2.2.2. Modified Simple Boundary Follower

**Figure 3:**
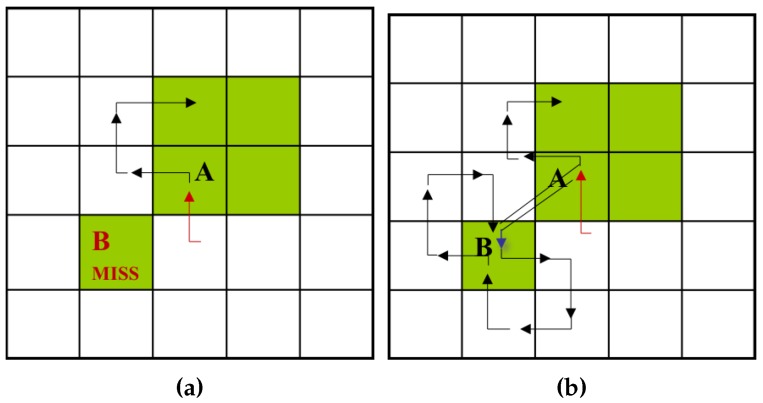
Detour of the inner-outer corner at the left-rear pixel (modified version of [3]). (**a**) Simple boundary follower (SBF); (**b**) modified SBF (MSBF).

SBF cannot trace an inner-outer corner pixel that is located at the left rear, and the modified SBF (MSBF) [3] was developed to trace these pixels. If the tracer is adjacent to the left-rear inner-outer corner, this condition implies that its left-rear pixel is black (object), and the left and rear pixels are white (background); the tracer will move to the left-rear pixel, and its direction is then changed toward the rear direction. After the movement, the tracer goes directly to the left pixel to avoid an infinite loop. Figure 3 shows examples of the SBF and MSBF paths for a left-rear direction inner-outer corner. In the case of the SBF, if the tracer is on pixel *A* with direction *N*, it misses pixel *B*. On the contrary, the MSBF can detour pixel *B*.

#### 2.2.3. Improved Simple Boundary Follower

The SBF and MSBF require movement operations for both contour and background pixels; therefore, time is wasted during movement on the background pixel, and they cannot trace the inner-corner pixel in front of the tracer [7,22]. Hence, we have proposed an improved SBF (ISBF) [7] that is based on our previous research aimed at overcoming these limitations. The ISBF has six cases for following contour pixels based on the local patterns of the contour pixels. The modified version [16] of ISBF is shown in Algorithm 2.

**Algorithm 2** Algorithm of the improved simple boundary follower.1:**procedure** ISBF2:    T(P,d)←S(P,d), where *P* is on black3:    **do**4:        **if** $P_{Left}$ = black **then**5:           // Case 4a: Left neighbor6:           $T\left(P,d\right)\leftarrow T\left(P_{Left},d_{Left}\right)$7:        **else**8:           **if** $P_{Left-Rear}$ = black and $P_{Rear}$ = white **then**9:               // Case 4b: Inner-outer corner at left-rear10:               $T\left(P,d\right)\leftarrow T\left(P_{Left-Rear},d_{Rear}\right)$11:           **else**12:               **if** $P_{Front-Left}$ = black **then**13:                   **if** $P_{Front}$ = black **then**14:                       // Case 4d: Inner corer at front15:                       $T\left(P,d\right)\leftarrow T\left(P_{Front},d\right)$16:                       $T\left(P,d\right)\leftarrow T\left(P_{Left},d\right)$17:                   **else**18:                       // Case 4c: Inner-outer corner at front-left19:                       $T\left(P,d\right)\leftarrow T\left(P_{Front-Left},d\right)$20:               **else** **if** $P_{Front}$ = black **then**21:                   // Case 4e: Front neighbor22:                   $T\left(P,d\right)\leftarrow T\left(P_{Front},d_{Right}\right)$23:               **else**24:                   // Case 4f: Outer corner25:                   $T\left(P,d\right)\leftarrow T\left(P,d_{Rear}\right)$26:    **while** T(P,d)≠S(P,d)

**Figure 4:**
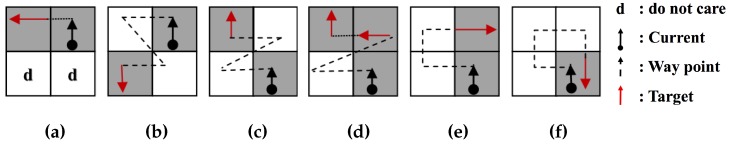
Contour cases of the improved SBF (ISBF) [16]: (**a**) left neighbor; (**b**) inner-outer corner at the left-rear; (**c**) inner-outer corner at the front-left; (**d**) inner corner at the front; (**e**) front neighbor; (**f**) outer corner. Reproduced with permission from [16].

Figure 4 represents the tracing path of the ISBF based on the local contour patterns. While the SBF cannot trace in Cases 4b and 4d and the MSBF cannot follow in Case 4d, the ISBF successfully traces in all of the cases. In the figure, the waypoint (dotted line) is subjected to a detection operation to determine whether it is black or white without employing a movement operation.

#### 2.2.4. Moore-Neighbor Tracing

Moore-neighbor tracing (MNT) finds the next contour pixel using eight connected chain codes with a clockwise sequence starting from the rear pixel of the tracer, *i.e.*, the tracer first moves toward the rear ($T(P_{Rear},d_{Rear})$) and finds the next clockwise contour pixel, such as the left-rear, left, font-left, front, front-right, right and rear-right pixels [20,22].

#### 2.2.5. Radial Sweep Algorithm

**Figure 5:**
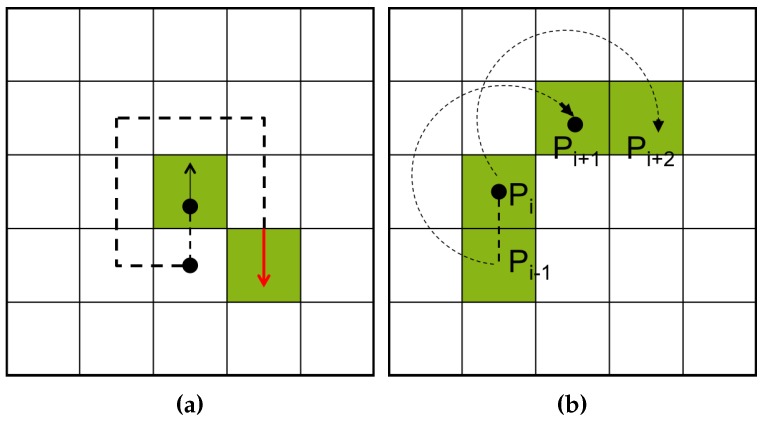
Contour-following sequence of Moore-neighbor tracing (MNT) and the radial sweep algorithm (RSA): (**a**) MNT [20]; (**b**) RSA [9].

The radial sweep algorithm (RSA) [9] is similar to MNT, but its tracer has no directional information. Therefore, it maintains two points, namely the previous pixel and current pixel for the initial tracing direction. Figure 5b illustrates an example of a tracing path obtained using RSA from $P_{i}$ to $P_{i+2}$. In the figure, the direction vector from $P_{i}$ to $P_{i-1}$ is first generated, and the tracer then searches for the next contour pixel using the previous pixel $P_{i-1}$ for the clockwise direction of the vector.

#### 2.2.6. Theo Pavlidis Algorithm

**Figure 6:**
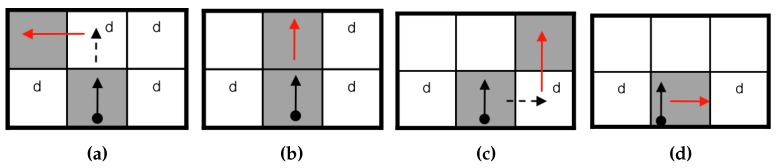
Contour-following sequence of the Theo Pavlidis algorithm (TPA) [16]. (**a**) Front-left contour; (**b**) front contour; (**c**) front-right contour; (**d**) rotation.

To determine the next contour pixel, the Theo Pavlidis algorithm (TPA) [8] considers only three adjacent pixels, e.g., front-left, front and front-right. If all three pixels are white, the tracer turns right. Figure 6 describes the sequence of this algorithm.

### 2.3. Conventional Data Compression and Restoration

**Figure 7:**
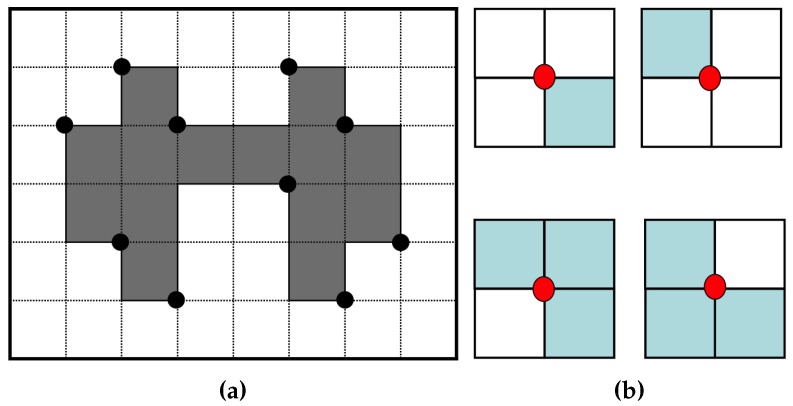
Representative points for data compression. (**a**) Representative points [17]; (**b**) cases of representative points.

The RD code method [17] comprises two major techniques. The first one traces the contour using a hybrid method that employs vertex following with run data, and it generates the corresponding RD codes. The other generates compressed contour data that can be used to restore the contour based on representative points and their corresponding RD codes. The representative points are selected from the vertices of contour pixels, and they are feature points of the contour. Moreover, the RD code can represent 10 local contour patterns and their corresponding following paths. Therefore, by saving the representative points and their corresponding RD codes instead of all of the contour points, we can reduce the memory size used to store the contour data. Figure 7a [17] gives an example of representative points. There are four types of representative points in the RD method, namely two outer corner points and two inner corner points, as shown in Figure 7b. Although this technique can save data in small files, it does not consider the inner-outer corner pixel.

## 3. Analysis of Conventional Contour-Following Algorithms

**Figure 8:**
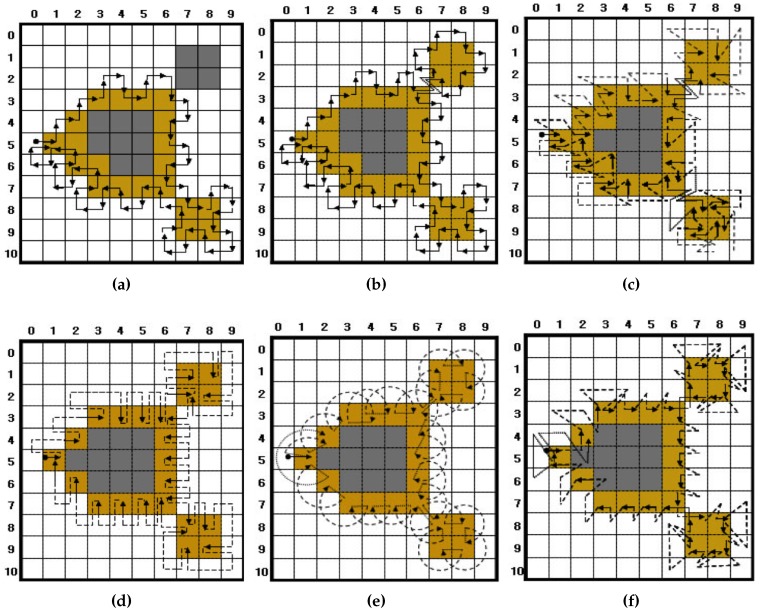
Comparison of conventional contour pixel-following algorithms: (**a**) SBF; (**b**) MSBF; (**c**) ISBF; (**d**) MNT; (**e**) RSA; (**f**) TPA.

Figure 8 illustrates the tracing results of the pixel-following methods based on the contour shown in Figure 1. In the figure, the arrow with an anchor is the tracer at the starting pixel, the solid arrow shows the movement operation of the tracer and the dotted line is the way point (detected pixel) for determining whether or not the pixel is a contour pixel for pixel following.

### 3.1. Pixel-Following Cases

As shown in Figure 8, ISBF traces most types of contour pixels, such as the inner corner, outer corner, inner-outer corner and straight-line pixels. In the case of the SBF, there are inconsistencies regarding tracing at the inner corner and inner-outer corner. For example, in the figure, the inner-corner pixel (3,6) and inner-outer corner pixel (7,8) are traced, but the inner corner (3,4) and inner-outer corner (7,9) pixels are missed. In the case of the MSBF, all of the inner-outer corner pixels are traced, but it has inconsistencies with regard to tracing the inner-corner pixel. Moreover, MNT, RSA and TPA have no problems with consistency, but they cannot trace the inner corners. Among these algorithms, the TPA can be easily changed to trace an inner-corner pixel, because it has waypoints on the inner corners, as shown in Figure 8f.

### 3.2. Start-Up Condition and Stopping Criteria

The pixel-following algorithm requires start and stop criteria to avoid incomplete or infinite contour tracing.

#### 3.2.1. Assumption for Start

Commonly, tracing starts when the tracer enters a black pixel from a white pixel. Therefore, at the start of tracing, the tracer must be placed on a black pixel, and its rear pixel $P_{Rear}$ should be white. Moreover, in the case of the MSBF and ISBF, if the $P_{Left-Rear}$ of the start pixel is an inner-outer corner pixel, it cannot trace all of the contour pixels using their stopping criteria [9,16]. In addition, TPA has to select a start pixel that has white pixels at the tracer positions $P_{Left}$, $P_{Left-Rear}$ and $P_{Right-Rear}$ [22]; otherwise, it cannot trace the left pixel of the contour.

#### 3.2.2. Stop Criterion

There are three methods for stopping the contour tracing [9,22]. The first method is Jacob’s stopping criterion [22], which terminates the trace when the tracer reenters the start pixel with an absolute direction that is the same as the start direction, *i.e.*, if the current tracer T(P,d) is the same as the start tracer S(P,d), the pixel following is terminated. SBF, MSBF and ISBF use this criterion, and their tracing terminates at the start pixel, as shown in Figure 8a–c. The second method uses the number of reentries to the start pixel. In Figure 8d–f, the tracers of MNT and TPA revisit the start pixel (1,5) with different directional information; therefore, they do not stop, but rather go to the next contour pixel if the first method is applied. For this reason, if a specified number of reentries, e.g., three or four times, is satisfied, the trace is terminated [9]. This method is sometimes not efficient, because it requires unnecessary tracing to be performed one or more times. The final method checks the trace route that is traced by the previous pixel and the current pixel of the tracer and determines whether it has already been passed. This method is used for RSA [9,22] because its tracer has only pixel-location information and no directional information. In other words, whenever the tracer enters the *i*-th contour pixel, the current pixel location $P_{i}$ is appended sequentially into the traced contour path. Moreover, if the traced path of $\left(P_{i-1},P_{i}\right)$ appears twice, the tracing is terminated. This method can be applied for all pixel-following methods, and it is simpler than the second method; however, it requires more operations than Jacob’s stopping criterion.

#### 3.2.3. Limitations of Conventional Pixel-Following Methods

The above-mentioned conventional pixel-following methods have certain limitations. First, some of the algorithms, such as SBF and MSBF, perform unnecessary movement operations on white pixels, as shown in Figure 8a,b. Second, not all of the algorithms can define the contour in the case of contour pixels; therefore, they cannot be a descriptive feature of the object and determine connectivity among objects. For example, in Figure 8b, MSBF detects (2,7) as the inner-outer corner pixel, but does not indicate (8,7) as an inner-outer corner, because the traced paths on the pixels are different. Moreover, MSBF also cannot determine the inner corner, outer corner and straight-line pixel, as is the case with SBF. In the case of ISBF, it determines the inner-outer corner, front-inner corner and front-straight line pixels, but cannot determine the left-inner corner, left-straight line and some of the outer-corner pixels, as shown in Figure 8c. Similarly, MNT and RSA cannot determine and detect the inner corners, and TPA cannot classify the contour pixels into the different types of contour pixels. Finally, the data size of the traced contour must be considered. The pixel-following methods save all of the pixel points; therefore, their data are larger than those of the RD code method.

## 4. Proposed Contour-Tracing Algorithm

In this section, we propose a novel pixel-following algorithm that traces contour pixels by considering their local patterns. Therefore, it can classify the contour pixel as inner corner, outer corner, inner-outer corner and straight-line contour types. Further, it can easily determine the next contour pixel. Moreover, it can determine and save representative points of an image, such as the RD code method, by using pixel following, but not using run-data-based following. In addition, the data can be restored to the original contour pixels without the RD code data.

### 4.1. Contour Following

#### 4.1.1. Assumptions for Start-Up and Stopping Criteria

The proposed algorithm runs under two assumptions for starting. One is the general condition for pixel following, where the rear pixel of the tracer on the start pixel is white. The other is that there is no left-rear inner-outer corner for the tracer at the start position, *i.e.*, if the rear and the left pixels are white and the rear-left pixel is black, the start pixel has to be changed. These are the same starting conditions as those used for MSBF and ISBF. Moreover, the stop criteria of the proposed algorithm is Jacob’s stopping criterion, and the tracer is always on a contour pixel.

#### 4.1.2. Procedures

**Figure 9:**
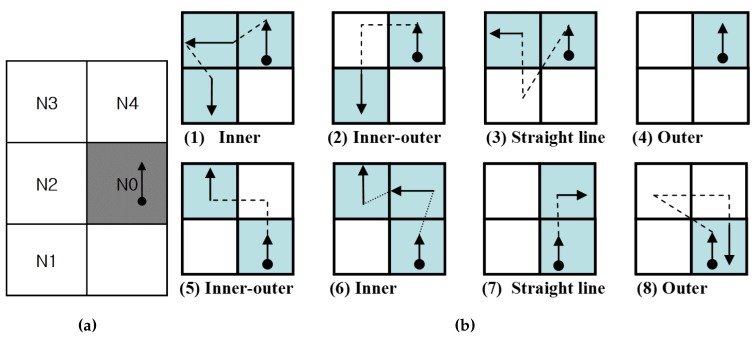
Contour tracing cases for the proposed contour-following algorithm. (**a**) Adjacent pixels; (**b**) contour cases.

The proposed algorithm has two stages. First, the tracer follows the contour pixel based on the intensities of the left-rear and left pixels. After that, the tracer follows the contour pixels according to the intensities of the front and front-left pixels. Figure 9 shows the contour tracing cases for the proposed contour-following algorithm. In the figure, the tracer is first on N0 and queries states of N1 and N2, as shown in Figure 9a, and then, the states determine the corresponding path to be traced from among Cases (1)–(4), as shown in Figure 9b. After Stage 1, the moved tracer queries states N3 and N4, and it then traces contour pixels along the corresponding path using the states from among Cases (5)–(8).

Hence, by using the proposed algorithm, the inner corners traced are considered as Case (1) or (6); the inner-outer corners are considered as Case (2) or (5); the outer corners are considered as Case (4) or (8); and the straight-line pixels are considered as the other cases. Therefore, all of the cases are easily classified using the algorithm.

#### 4.1.3. States

**Figure 10:**
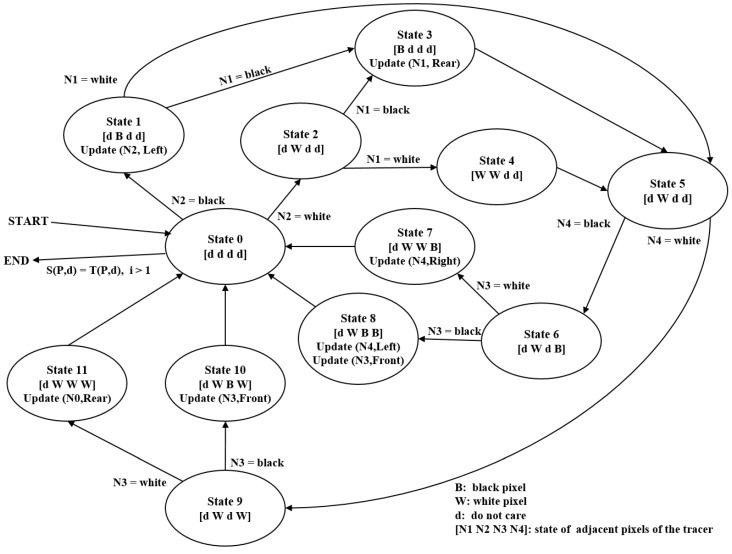
State transition of automation for the proposed algorithm.

Figure 10 describes the state transition for the automation of the proposed algorithm. In the figure, the start and termination occur at State 0. The first stage runs using States 1–4, and it then transits to State 5. The second stage continues from State 5 using States 6–11, and it then transits to State 0. For example, Case (1) in Figure 9b can be executed using the transit sequence State 1, State 3 and State 5.

In Figure 10, [N1N2N3N4] represents the intensity vector of the tracer’s four adjacent pixels shown in Figure 9a. In the vector, *d* implies “do not care”; *B* represents a contour pixel (black) and *W* represents the background pixel (white). Moreover, update (P,d) refers to the movement operation, where *P* is the new contour-pixel location and *d* is the new directional information for the tracer. In States 1 and 3, the updates occur based on the tracer of State 0, and other updates are based on the tracer information in State 5.

#### 4.1.4. Characteristics of the Proposed Algorithm

We designed the proposed algorithm based on two stages with two major goals. First, the check operation for stopping occurs only at State 0; therefore, the number of checking operations on black pixels is reduced. In other words, the proposed algorithm verifies that the check operation occurs when only the tracer has a white rear pixel. This is more efficient when compared to the check operation that occurs for every contour pixel, because its start condition and stop criterion also satisfy the condition that the rear pixel of the tracer on the start pixel should be white. Therefore, the transition of Stage 2 to States 6–11 for processing Cases (5)–(8) is designed such that it can be returned to State 0 only if the tracer has a white rear pixel, and it reduces any unnecessary operations that are required to stop the checking. Besides, the tracers of Cases (2) and (4) have a white rear pixel after the movement, but their end conditions are not considered. In Case (2), at the start, the tracer avoids the inner-outer corner pixels as the start and end pixel. Moreover, in Case (4), the tracer has no update; therefore, it is unnecessary to perform the check operation twice.

**Figure 11:**
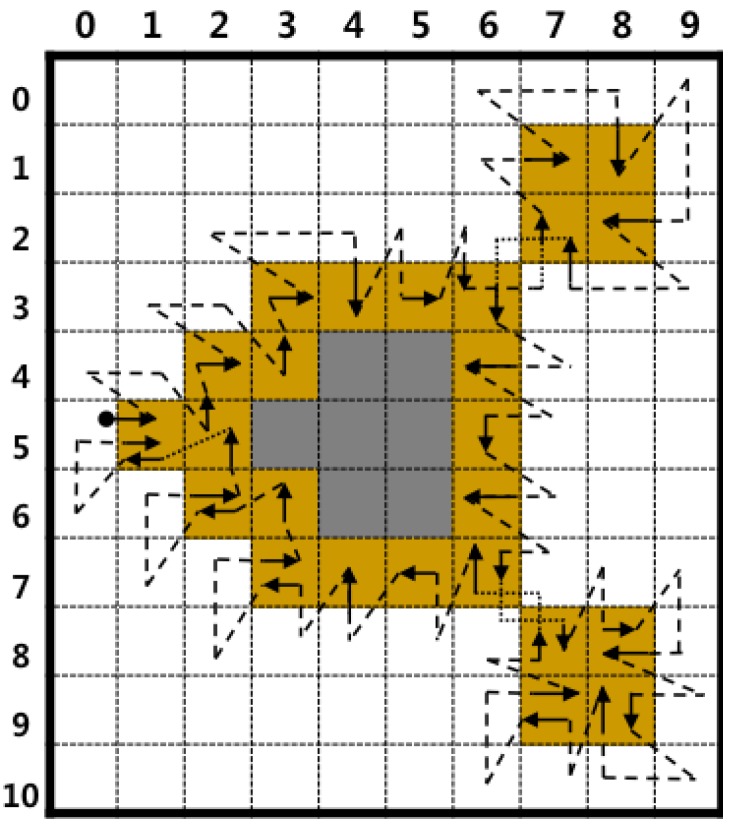
Result of contour tracing using the proposed algorithm.

Second, the proposed algorithm eliminates some of the redundant operations that are used to detect white pixels. The conventional algorithms do not consider white pixels in the previous path; therefore, they sometimes re-detect white pixels on the previous tracing during the current tracing. For example, in Figure 8, the white pixel at (4,2) is detected twice while determining contour pixels, such as (4,3) and (5,3). Moreover, RSA detects (4,2) three times while determining (4,3), (5,3) and (6,3). On the contrary, the proposed algorithm has two stages, and its second stage avoids the previous path. Figure 11 shows the contour-tracing results obtained for the proposed algorithm, and it detects (4,2) once for contour tracing. Moreover, the figure shows that the proposed algorithm has fewer operations on the white pixels when compared to conventional pixel-following methods, as shown in Figure 8, and it traces all types of contours.

The pseudocode of the proposed algorithm is shown in Algorithm 3.

Table 2 describes the tracing results that were obtained by using the proposed algorithm up to the stage at which the tracer enters the 11th pixel. The code represents the contour pixel type, and it is classified automatically during tracing.

**Algorithm 3** Procedure of the proposed tracer.1:**procedure** Proposed_Tracer2:    T(P,d)←S(P,d), where *P* is on black, $P_{Rear}$ is on white and i←13:    // Whenever T(P,d) is updated, *i* increases by1 and $T(p^{\prime },d^{\prime })$ is saved automatically4:    **do**5:        // Stage 16:        **if** $P_{Left-Rear}$ = black **then**7:           **if** $P_{Left}$ = black **then**8:               // Case 19:               $T\left(P,d\right)\leftarrow T\left(P_{Left},d_{Left}\right)$ and Code(i)←“Inner”10:               $T\left(P,d\right)\leftarrow T\left(P_{Left},d_{Left}\right)$11:           **else**12:               // Case 213:               Code(i)←“Inner−outer”14:               $T\left(P,d\right)\leftarrow T\left(P_{Left-Rear},d_{Rear}\right)$ and Code(i)←“Inner−outer”15:        **else**16:           **if** $P_{Left}$ = black **then**17:               // Case 318:               $T\left(P,d\right)\leftarrow T\left(P_{Left},d_{Left}\right)$ and Code(i)←“Straight”19:           **else**20:               // Case 421:               Code(i)←“Outer”   22:        // Stage 223:        **if** $P_{Front-Left}$ = black **then**24:           **if** $P_{Front}$ = black **then**25:               // Case 626:               $T\left(P,d\right)\leftarrow T\left(P_{Front},d_{Left}\right)$ and Code(i)←“Inner”27:               $T\left(P,d\right)\leftarrow T\left(P_{Front},d_{Right}\right)$28:           **else**29:               // Case 530:               Code(i)←“Inner−outer”31:               $T\left(P,d\right)\leftarrow T\left(P_{Front-Left},d\right)$ and Code(i)←“Inner−outer”32:        **else** **if** $P_{Front}$ = black **then**33:           // Case 734:           $T\left(P,d\right)\leftarrow T\left(P_{Front},d_{Right}\right)$35:        **else**36:           // Case 837:           $T\left(P,d\right)\leftarrow T\left(P,d_{Rear}\right)$ and i←i−1 and Code(i)←“Outer”38:    **while** T(P,d)≠S(P,d)

In the table, Code(i) represents only one code, the contour pixel type per contour pixel, but it can have several codes. For example, there is an outer-corner pixel and an inner-outer corner pixel.

### 4.2. Data Compression and Restoration

The proposed algorithm saves representation points and the inner-outer corner points in the form of compressed data in order to reduce the data size. The representation points are feature points that are used for storing and restoring contour pixels, while the inner-outer corner points are used for accurately restoring the inner-outer corner pixels.

#### 4.2.1. Data Structure

**Figure 12:**
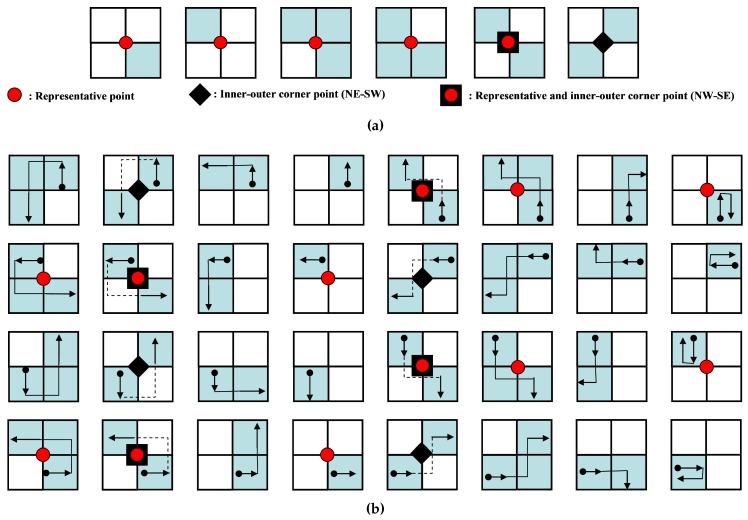
Contour pixel reconstruction. (**a**) Representative points and inner-outer corner points; (**b**) cases of the proposed algorithm.

The representative points and inner-outer corner points are represented as vertices of contour pixels. Figure 12a shows the point types of the proposed algorithm. There are seven types of representative points. They are two outer corners, two inner corners and one inner-outer corner. In addition, there are two types of inner-outer corner point. Figure 12b illustrates all cases of contour tracing for the proposed algorithm and their corresponding representative points and inner-outer corner points.

These points are saved as a sequence during contour tracing. If the *i*-th representative point $R_{i}$ is equivalent to $\left(r_{i,x},r_{i,y}\right)$, then the set of representative points *R* is given by $\left\{R_{0},R_{1},R_{2},\cdots ,R_{n-1}\right\}$ and $R_{0}=R_{n}$ because the starting and ending points are the same. Similarly, if $C_{j}$ is the *j*-th inner-outer corner point, it can be represented using its coordinate and its type. The type of point $C_{j,T}$ is assigned to be NW−SE or NE−SW, as shown in Figure 12a. Table 3 shows the data structure for data compression and the restoration of the contour pixels using the proposed algorithm.

#### 4.2.2. Contour Pixel Restoration

For the restoration, we propose a restoration algorithm comprising two stages, namely contour restoration and inner-outer corner restoration.

##### Contour Restoration with Representation Points

The sequence of representative points is important for the reconstruction of the contour pixels, because it represents the contour-tracing sequence. Hence, by using the sequence of representative points and the relative location between adjacent representative points in the representative point table, the contour can be restored easily. If there are two sequential representative points $R_{i}$ and $R_{i+1}$, $\Lambda (R_{i},R_{i+1})$, which is the relative position classifier from $R_{i}$ to $R_{i+1}$, can be described as:

(1) $$\Lambda \left(R_{i},R_{i+1}\right)=\left\{\begin{array}{c} NE\;\text{where}\;r_{i,x}<r_{i+1,x}\;\text{and}\;r_{i,y}>r_{i+1,y}\\ SE\;\text{where}\;r_{i,x}<r_{i+1,x}\;\text{and}\;r_{i,y}<r_{i+1,y}\\ NW\;\text{where}\;r_{i,x}>r_{i+1,x}\;\text{and}\;r_{i,y}>r_{i+1,y}\\ SW\;\text{where}\;r_{i,x}>r_{i+1,x}\;\text{and}\;r_{i,y}<r_{i+1,y} \end{array}\right.$$

**Figure 13:**
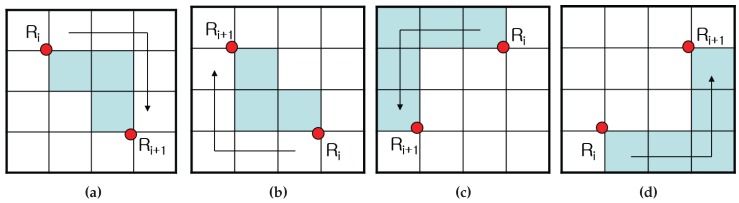
Contour pixel reconstruction. (**a**) SE; (**b**) NW; (**c**) SW; (**d**) NE.

Figure 13 shows the contour-pixel reconstruction methods using the relative positions from $R_{i}$ to $R_{i+1}$. In the case of SE or NW, the contour pixels are filled in a clockwise manner, while in the other cases, these pixels are filled in a counterclockwise manner.

**Figure 14:**
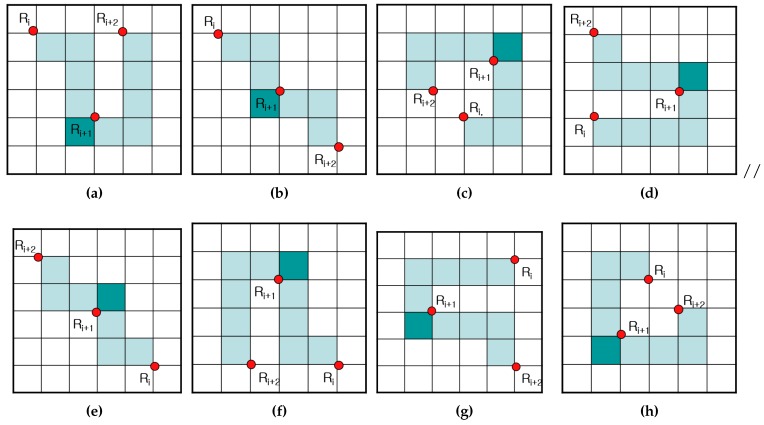
Restoration cases for different sequences of representation points. (**a**–**h**) Case 1–8.

The methods in Figure 13 are the basic methods employed to restore the contour, but they are problematic when used to reconstruct the inner-corner pixel using three or more representative points. Table 4 and Figure 14 show cases of the missing inner-corner pixels using sequential representative points $R_{i}$, $R_{i+1}$ and $R_{i+2}$.

**Figure 15:**
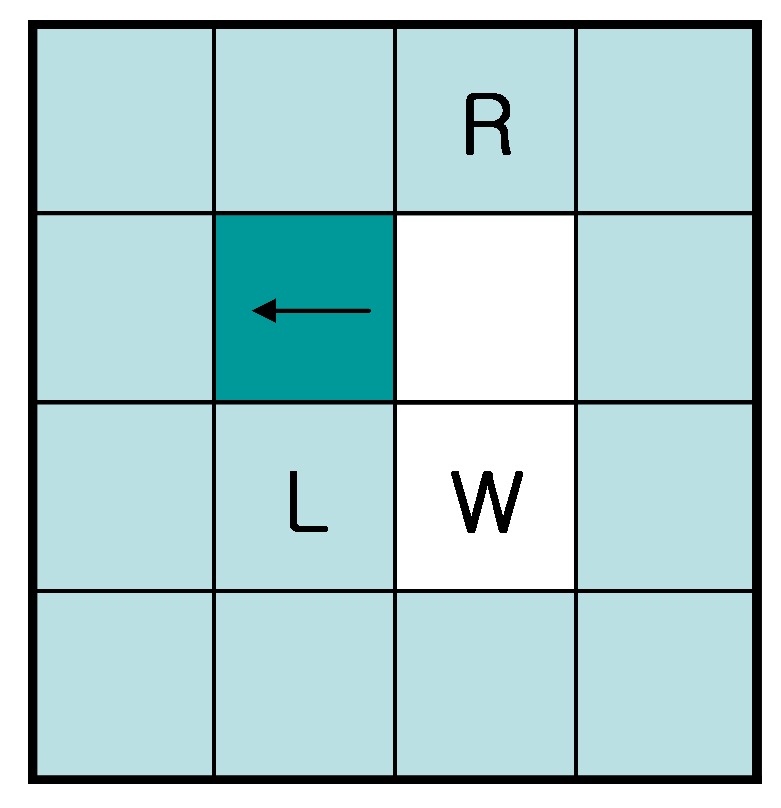
Problem with TPA. TPA must start with white left (L), left-rear (W) and right-rear (R) pixels. However, generic inner contours cannot satisfy these criteria.

As shown in Figure 15, the three representative points cannot restore the inner-corner pixel $P_{m}$. Therefore, if the three sequential points form one of the cases in Table 4, $P_{m}$ of the middle representative point $R_{i+1}$ must be filled with a dark color.

This reconstruction method is different from the restoration approach in the RD code method because the proposed method does not need the RD code, but requires only representative points, and their saved sequence naturally replaces the RD code. Therefore, it requires a smaller memory size when compared to the RD code method.

##### Inner-Outer Corner Restoration

The restored contour obtained using only the representative points has no inner-outer corners because the inner-outer corner is not considered. For this reason, if there are inner-outer corner points in the data table, as shown in Table 4, the inner-outer corners are generated using the data $C_{j}$ with their point coordinates and types. If a pixel restored using the representative points is o(x,y) and *O* is the restored contour, the function of the inner-outer corner restoration, $R_{IO}$, can be obtained as:

(2) $$R_{IO}=\left\{\begin{array}{c} O-o\left(c_{j,x}-0.5,c_{j,y}-0.5\right)-o\left(c_{j,x}+0.5,c_{j,y}+0.5\right),\text{where}\;c_{j,T}=“NW-SE”\\ O-o\left(c_{j,x}-0.5,c_{j,y}+0.5\right)-o\left(c_{j,x}+0.5,c_{j,y}-0.5\right),\text{where}\;c_{j,T}=“NE-SW” \end{array}\right.$$

## 5. Experimental Result

To compare the proposed algorithm to conventional algorithms, we perform an experiment to determine their accuracy, speed and stored data size. Table 5 shows the experimental environment.

We experimented on nine CCITT (Consultative Committee for International Telephony and Telegraphy) standard fax images with 200 dots per inch (dpi) [17]. All of these images have 1728×2339 pixels and a file size of 11,842 KB. Table 6 shows the document type of these images and the total number of contour pixels. We used these large-sized images because they have various types of contours, which is useful when comparing the efficiencies with regard to parameters, such as processing time and the accuracy of the trace results of the contour-tracing algorithms.

In order to compare the proposed algorithm to the conventional algorithms, we used the experimental method described in [18] to determine the start pixels of the outer and inner contours of the images. In other words, whenever any untraced contour pixel is searched using a raster scan from the left top to the right bottom of the images, this pixel is regarded as the start pixel, and the tracer starts contour tracing. If the contour is an outer contour, the tracer’s initial direction is assigned as east (“E”). On the contrary, in the case of the inner contour of an object, e.g., “e,” “p,” “q,” “R” and “o,” the tracer’s initial direction is assigned as west (“W”).

In the experiments, we did not consider the TPA because the initial condition of the TPA is that it must start with white left (“L”), left-rear (“W”) and right-rear (“R”) pixels [22], which is not satisfied in some of the inner contours. Figure 15 shows an example of this violation. In our experiments, there were many cases for which these conditions were not satisfied. Therefore, we could not perform identical experiments, and meaningful data were not obtained for comparing the trace results with those of the other methods.

### 5.1. Accuracy

The accuracy of contour tracing involves determining how accurately the tracing algorithm traces, and we measure it by counting the number of pixels traced. First, we apply each algorithm to the test images and mark the tracing on the images. Then, we count all of the marked contour pixels in the images. Therefore, even if a pixel is traced several times, it is counted only once. Table 7 shows the results of the comparison between the proposed algorithm and the conventional ones. In the table, “total number” implies the total number of ground truth contour pixels, including the inner corner, outer corner, inner-outer corner and straight-line pixels. The ground truth pixels are counted that are adjacent to the background pixels. In this result, MNT and RSA traced the least number of pixels as contours, because they could not trace the inner-corner pixels. SBF has inconsistencies with regard to the inner-outer corner and inner-corner types. Therefore, it traced fewer pixels when compared to ISBF and the proposed algorithm. Further, MSBF has inner-corner inconsistencies that are similar to those of SBF, and MSBF traced fewer pixels when compared to the proposed algorithm and ISBF. The proposed algorithm shows that 99.5% of the total contour pixels were found to be the same as those in the case of ISBF, and it has the maximum total number of traced contour pixels. In conclusion, the proposed algorithm produced the best results with regard to tracing accuracy.

**Figure 16:**
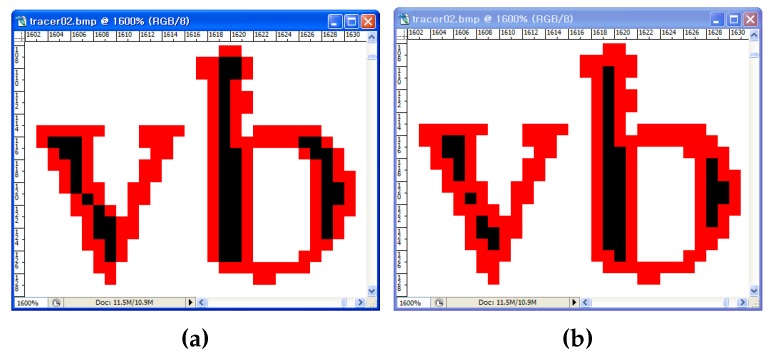
Visual comparison of two contour-tracing methods. (**a**) MSBF; (**b**) the proposed method.

**Figure 17:**
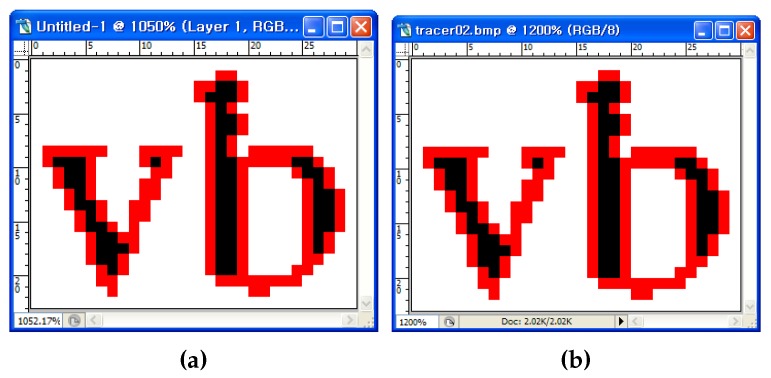
Visual comparison of two contour-tracing methods. (**a**) MNT; (**b**) the proposed method (without inner corners).

Figure 16 shows the traced images resulting from MSBF and the proposed algorithm. As shown in Figure 16a, MSBF was not able to trace some of the inner corner pixels, but our proposed method (Figure 16b) was able to trace all of the corner-pixel types without any inconsistency. Moreover, as the proposed algorithm can classify each corner type, it can trace the selected type of contour pixels by omitting some cases, as shown in Figure 9b. For example, if we remove the tracing Cases (1) and (6) from the other cases, we can obtain a result without inner-corner tracing, and it is the same as the result of MNT and RSA. Figure 17b shows an image that was traced using the proposed algorithm without inner corners, and it shows that the image is consistently traced without inner corners.

### 5.2. Speed

In order to measure the tracing time for each algorithm, we performed each algorithm 20 times per image and calculated the average time. We used the cv2.getTickCount() function supported by OpenCV 3.0.0 to measure the processing time. Table 8 shows the average processing time of each algorithm used for tracing the images and a linear model for estimating the process time as the number of traced pixels increases using the least-square estimation (LSE) method. In the table, we obtain the average processing time per traced contour pixel by dividing the total processing time by the total number of traced contour pixels.

**Figure 18:**
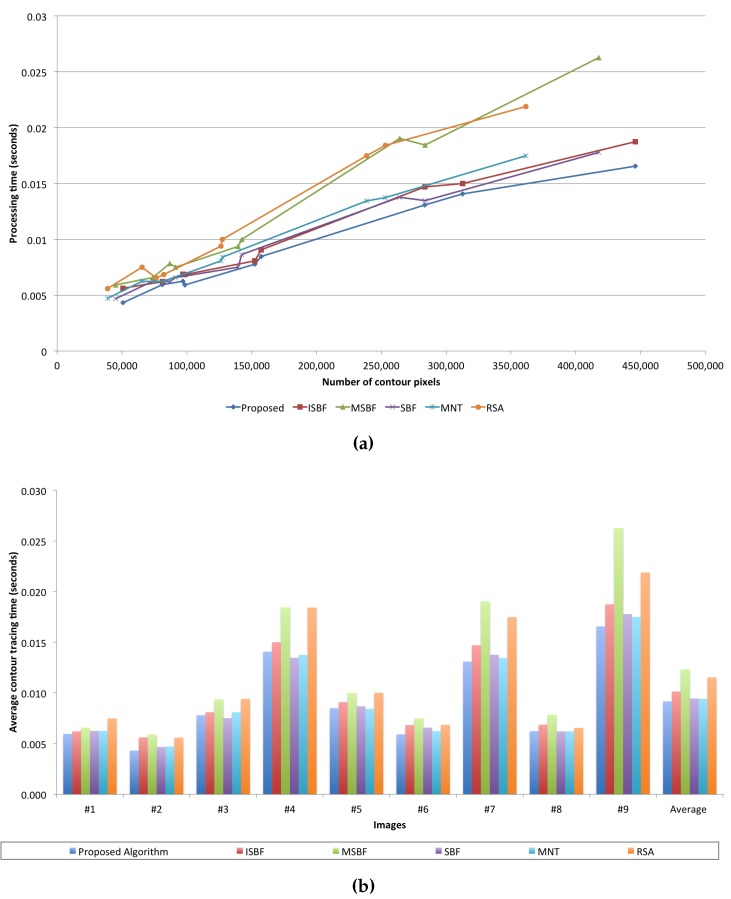
Comparison of tracing times of the contour-tracing algorithms. (**a**) Processing time *vs.* the number of contour pixels; (**b**) average contour tracing time *vs.* images.

Figure 18 illustrates a graph that uses data from Table 7. As shown in Figure 18, the proposed algorithm has the best performance, *i.e.*, it had the least average processing time and showed the smallest increase in the ratio of the process time to the number of traced contour pixels, as shown in the LSE. In particular, although the proposed algorithm traced most of the contour pixels in each image, it has the best or a good performance when compared to the conventional algorithms. Furthermore, the proposed algorithm shows better standard deviation results than the conventional algorithms. Therefore, the proposed algorithm has better performance than the other algorithms for the number of traced contour pixels and the processing time.

### 5.3. Reduced Memory

The proposed algorithm does not save all of the contour pixels, but it saves only the representative points and the inner-outer corner pixels. Table 9 shows the data size acquired from the above experiments performed using CCITT standard fax images. It shows the data sizes of traced contour pixels and their compressed data. The number of traced contour pixels (*A*), which are the same results from Table 7, and *C* and *D* in the table indicate the number of representative points and inner-outer corner points of the traced contour pixels. *A* and *C* are the number of (x,y) coordinates, and *D* represents the number of inner-outer corners that comprise (x,y) coordinates and the type of inner-outer corner. The benefit of storing only the representative points based on the vertex of the contour pixel is that it can significantly reduce the data size. The experimental results obtained show that the proposed algorithm reduced the data size to 19%–60% of the memory used when all of the contour pixels were stored, as shown in Table 9.

### 5.4. Restoration

**Figure 19:**
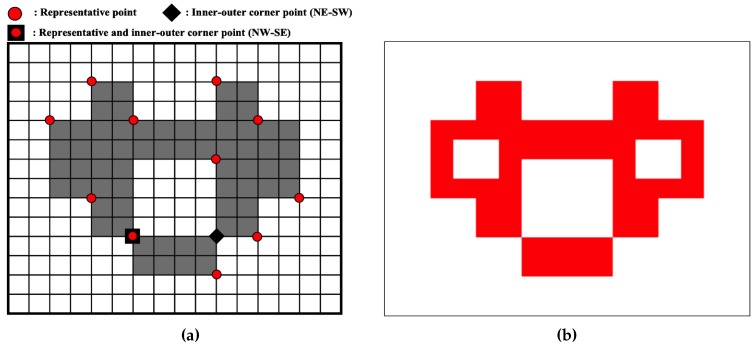
Example of the restoration of contour pixels. (**a**) The original image and its saved points for restoration; (**b**) restoration by the saved data.

Figure 19 shows an example of the retrieval points and a restoration result obtained using the proposed restoration algorithm. Figure 19a is an example image that has all of the contour pixel types, and it depicts the representative points and inner-outer corner points for contour description and restoration. This image has two contours, namely an outer contour and an inner contour that includes two inner-outer corners. Table 10 describes these data, and Figure 19b shows the restoration results, which were retrieved using the data from Table 10. In the figure, the restored contour accurately represents the original contour pixels.

**Figure 20:**
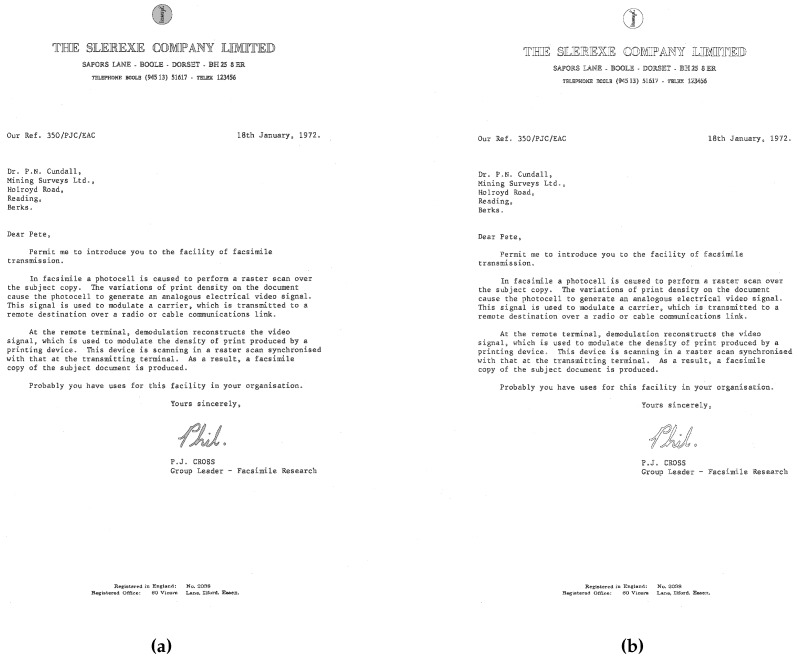
Result of the experiment for CCITT #1. Contour pixels are shown in black, original image pixels in grey. (**a**) Result of contour tracing; (**b**) result of contour restoration.

Figure 20 shows the result obtained for the CCITT #1 image using the proposed restoration algorithm. Figure 20a represents the contour-tracing result, and Figure 20b depicts the result of the restoration from the compressed contour data. To verify the identity, we compared the contour pixels of the two images and found that they are identical with regard to the number of contour pixels and the pixel coordinates, *i.e.*, the contour pixels in the restoration result are the same as the original contour pixels. As shown in Figure 19 and Figure 20, these experiments proved that the proposed algorithm could trace the inner and outer contours. Further, it was able to store the results using less memory by storing only the representative points and inner-outer corner points instead of all of the contour pixels; moreover, it could correctly restore all of the contour pixels from the compressed data. Besides, as shown in [17], compressed data based on vertex contours guarantee precise enlarging.

### 5.5. Limitations

**Figure 21:**
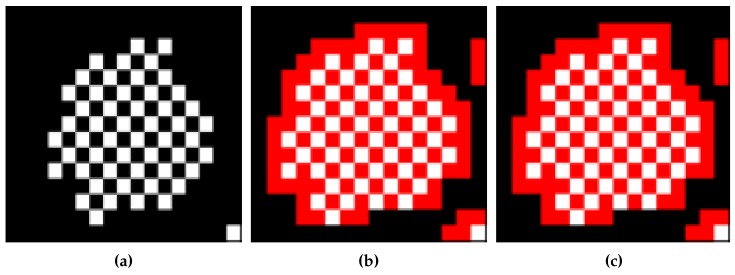
Example of untraced contour pixels caused by missing starting pixel CCITT Image #9 From (1093,1766) to (1108,1780). (**a**) Original image; (**b**) traced by ISBF; (**c**) traced by the proposed algorithm.

In the experiments, there were missing contour pixels that did not satisfy the experimental conditions described in [17]. In Figure 21b,c, there are eight untraced contour pixels because the horizontal scan line cannot find a starting pixel for the contour under certain conditions. In other words, because the scan line seeks an untraced black pixel with an adjacent white pixel on the horizontal line, if the untraced contour pixel that is between two black pixels in the horizontal direction has an adjacent white pixel in the vertical and/or diagonal direction, the untraced contour pixel cannot be considered as the starting pixel. Therefore, as shown in Table 7, the missing contour pixels remain after running the proposed algorithm, and the untraced contour pixels of other algorithms are included in the missing contour pixels for the same reason. In particular, Image #9 has the largest number of missing contour pixels because it has many one-pixel-sized chessboard patterns that comprise inner-outer corner pixels. The chessboard pattern consists of one-pixel-sized inner-outer corner pixels, which tend to cause the missing start-pixel problem.

**Figure 22:**
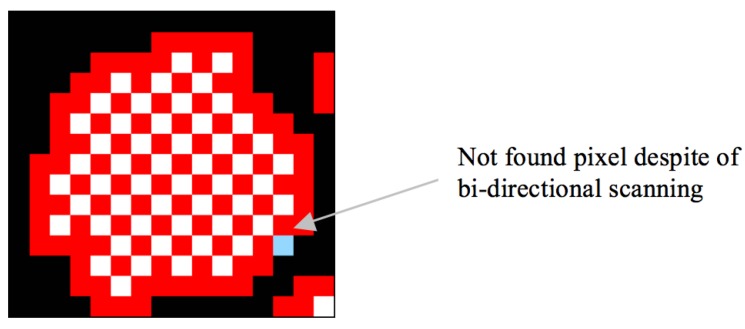
Result of the proposed algorithm by using bidirectional scanning.

To overcome the problem, we applied an eight-connection mask to the images to obtain the starting pixel, but the mask required many operations. In other words, we attempted to measure the performance of multi-direction scanning in order to eliminate the missing contour-pixel problem by using vertical and horizontal scans instead of an eight-connection mask operation. Table 11 shows the increase in the number of pixels traced using bidirectional scanning, and Table 12 describes the processing time for this method. Moreover, Figure 22 shows the tracing result that was obtained using the proposed algorithm based on bidirectional scanning, and it shows that seven of the missing pixels are traced, but one diagonal connective-contour pixel remained untraced.

In the above tables, bidirectional scanning slightly increases the number of traced contour pixels, but their processing time increases significantly. Moreover, the proposed algorithm shows acceptable performance in terms of accuracy (99.5%), although we performed only unidirectional scanning. Hence, unidirectional scanning based on the proposed algorithm is sufficient for the application to contour tracing under the condition that relatively few objects are present, and we performed real-time tracing, such as AR, MR and recognition-image-based code on small-scale images, such as those in a mobile computing environment.

## 6. Conclusions

In this paper, we proposed a contour-tracing algorithm to trace contours in low-performance devices, such as mobile phones, PDAs and embedded devices that have a processor with limited computational capability and a small memory capacity. The proposed algorithm traces contour pixels based on the pixel-following method, and it can also convert the contour information to compressed data and accurately restore it to the original contour using the vertex-following method. The proposed algorithm repeatedly executes the two tracing stages. First, the tracer moves to the next pixel based on its left and left-rear pixels. Next, it moves based on the front and front-left pixels. With these two tracing stages, the proposed algorithm extracts two contiguous contour pixels together. Because the proposed algorithm traces contiguous pixel pairs in a single step, there are more possible cases of the form a contour can take. Therefore, the classification of the contour is more complicated than the conventional algorithms. On the other hand, this classification actually takes less time to compute, because it reduces the duplicated detection of the background pixels. Moreover, based on the classified cases, we can determine the representative points and the inner-outer corner points that are based on the coordinates of the vertices, and we can store the contour data as points in order to reduce the data size. In addition, we proposed a restoration algorithm to retrieve all of the contour pixels from the representative points and the inner-outer corner points. The proposed algorithm performs accurate restoration, and it can restore the inner-outer corners that were not considered in conventional algorithms, such as the RD code method and the PXY method. Another characteristic of the proposed algorithm is that it can trace the desired type of connectivity because it is able to distinguish between the different types of connections of the contour pixels. For example, the proposed algorithm may trace without inner corners, which is similar to the performances of MNT and RSA.

We performed experiments with regard to three aspects: accuracy, speed and saving data. From the experiment results, the proposed algorithm had the best performance with regard to the accuracy of contour tracing, *i.e.*, of all the algorithms, it traced the largest number of contour pixels. Moreover, it had the smallest average processing time per contour pixel and good performance with respect to the processing time of each image and the LSE. For this reason, it is considered to have reasonable performance, and based on its accuracy and processing time, it is regarded as the best of the different algorithms. In addition to the accuracy and speed, the proposed algorithm exhibited good performance with regard to the memory consumption. It stored only the representative points and inner-outer corner points, thus reducing the memory consumption. Besides, the proposed restoration algorithm successfully retrieved all of the contour pixels from the compressed data. Therefore, the proposed algorithm shows improved accuracy and fast processing of contour tracing, low memory consumption for saving the contour and good restoration ability.

## Figures and Tables

**Table 1:** Comparison of contour-following algorithms.

	Pixel-Following Method	Vertex-Following Method	Run-Data-Based Following Method
Traced object	Contour pixel	Vertex of contour (pixel corner)	Run-data
Data construction	Coordinates of contour pixels obtained using traced sequence (automatically)	Coordinates of vertices of contour pixels obtained using traced sequence (automatically)	All run-data of image and run-following relationship data (additive operation for calculating the relationship between adjacent run-data horizontally)
Adaptive application [17]	Small-scale image Slow trace is allowed	Small-scale image Slow trace is allowed	Large-scale image, such as document recognition

**Table 2:** Result table of the proposed contour tracing.

Sequence(i)	P		Code(i)
	*x*	*y*	
1	1	5	Outer
2	2	5	Inner
3	2	4	Outer
4	3	4	Inner
5	3	3	Outer
6	4	3	Straight
7	5	3	Straight
8	6	3	Inner-outer
9	7	2	Inner-outer
10	7	1	Outer
11	8	1	

**Table 3:** Data structure of the proposed contour tracer.

Representative Points ($R_{i}$)		Inner-Outer Corner ($C_{i}$)		
*x*	*y*	*x*	*y*	Type
$r_{1,x}$	$r_{1,y}$	$C_{1,x}$	$C_{1,y}$	$C_{1,T}$
$r_{2,x}$	$r_{2,y}$	$C_{2,x}$	$C_{2,y}$	$C_{2,T}$
...	...	...	...	...

**Table 4:** Examples of the inner corner missing.

Case	$\left(R_{i},R_{i+1}\right)$	$\left(R_{i+1},R_{i+2}\right)$
1	SE	NE
2	SE	SE
3	NE	SW
4	NE	NW
5	NW	NW
6	NW	SW
7	SW	SE
8	SW	NE

**Table 5:** Experimental environment.

	Desktop
CPU	Intel® Core™ i7-2600K CPU @3.40 GHz
Memory	14.0 GB
HDD	Seagate 1 TB Momentus ST1000LM024
OS	Microsoft Windows 7
Development	Microsoft Visual Studio 2013

**Table 6:** CCITT (Consultative Committee for International Telephony and Telegraphy) fax standard images.

Index	Type	Total Number of Contour Pixels
1	Business letter	81,189
2	Circuit diagram	50,825
3	Sales order table	152,489
4	French document	312,812
5	Technical paper	157,377
6	Technical graph	98,579
7	Japanese document	283,717
8	Handwritten memo	97,031
9	Facsimile test chart	453,721

**Table 7:** Comparison of traced contour pixels.

Image	Total Number	Proposed		ISBF		MSBF		SBF		MNT		RSA	
		Number	%	Number	%	Number	%	Number	%	Number	%	Number	%
#1	81,189	81,188	100.0	81,188	100.0	73,743	90.8	73,613	90.7	65,503	80.7	65,503	80.7
#2	50,825	50,824	100.0	50,824	100.0	45,003	88.5	45,003	88.5	38,819	76.4	38,819	76.4
#3	152,489	152,487	100.0	152,487	100.0	139,589	91.5	139,589	91.5	126,414	82.9	126,414	82.9
#4	312,812	312,812	100.0	312,812	100.0	283,709	90.7	283,712	90.7	253,169	80.9	253,169	80.9
#5	157,377	157,374	100.0	157,374	100.0	142,447	90.5	142,453	90.5	127,306	80.9	127,306	80.9
#6	98,579	98,566	100.0	98,566	100.0	91,176	92.5	91,174	92.5	82,239	83.4	82,239	83.4
#7	283,717	283,551	99.9	283,551	99.9	264,108	93.1	264,067	93.1	238,533	84.1	238,533	84.1
#8	97,031	97,015	100.0	97,015	100.0	86,822	89.5	86,822	89.5	76,251	78.6	76,251	78.6
#9	453,721	445,975	98.3	445,972	98.3	417,687	92.1	417,735	92.1	361,439	79.7	361,439	79.7
Total	1,687,740	1,679,792	99.5	1,679,789	99.5	1,544,284	91.5	1,544,168	91.5	1,369,673	81.2	1,369,673	81.2

**Table 8:** Speed experimental result (units: seconds).

Image	Proposed Algorithm		ISBF		MSBF		SBF		MNT		RSA	
	Mean	SD	Mean	SD	Mean	SD	Mean	SD	Mean	SD	Mean	SD
#1	0.00596	0.00032	0.00622	0.00069	0.00658	0.00074	0.00626	0.00060	0.00626	0.00054	0.00750	0.00058
#2	0.00432	0.00040	0.00562	0.00047	0.00592	0.00005	0.00468	0.00044	0.00472	0.00026	0.00560	0.00034
#3	0.00778	0.00046	0.00808	0.00036	0.00936	0.00085	0.00752	0.00069	0.00808	0.00033	0.00940	0.00066
#4	0.01406	0.00006	0.01500	0.00099	0.01844	0.00100	0.01346	0.00006	0.01374	0.00075	0.01842	0.00074
#5	0.00848	0.00038	0.00908	0.00080	0.01000	0.00086	0.00866	0.00054	0.00842	0.00075	0.01000	0.00065
#6	0.00592	0.00056	0.00684	0.00074	0.00750	0.00070	0.00658	0.00062	0.00624	0.00072	0.00686	0.00058
#7	0.01308	0.00068	0.01470	0.00096	0.01904	0.00110	0.01376	0.00091	0.01344	0.00058	0.01748	0.00072
#8	0.00624	0.00067	0.00688	0.00062	0.00784	0.00065	0.00622	0.00068	0.00622	0.00068	0.00656	0.00061
#9	0.01656	0.00079	0.01874	0.00063	0.02628	0.00053	0.01778	0.00086	0.01750	0.00089	0.02188	0.00093
Average	0.00916		0.01013		0.01233		0.00944		0.00940		0.01152	
Average time per traced contour pixel	$4.91\times 10^{-8}$		$5.43\times 10^{-8}$		$7.19\times 10^{-8}$		$5.50\times 10^{-8}$		$6.18\times 10^{-8}$		$7.57\times 10^{-8}$	
LSE	$3.23\times 10^{-8}+0.0031$		$3.57\times 10^{-8}+0.0035$		$5.74\times 10^{-8}+0.0025$		$3.58\times 10^{-8}+0.0033$		$4.06\times 10^{-8}+0.0032$		$5.54\times 10^{-8}+0.0031$	
R-square	0.98117		0.98544		0.98838		0.98685		0.99461		0.97615	

Results of best or faster speed/smaller standard deviation than the proposed algorithm are marked with a shadow.

**Table 9:** Comparison between total contour pixels and representative points.

	Entire Contour Pixels		Compressed Data			Ratio (%) (E/B×100)
	Number of contour pixels (*A*)	Data size (B=A×2)	Number of representative points (*C*)	Number of inner outer corner points (*D*)	Data size (E=C×2+D×3)	
#1	81,188	162,376	21,206	61	42,595	26.23
#2	50,824	101,648	12,695	21	25,453	25.04
#3	152,487	304,974	30,181	53	60,521	19.84
#4	312,812	625,624	86,953	442	175,232	28.01
#5	157,374	314,748	37,386	113	75,111	23.86
#6	98,566	197,132	18,464	104	37,240	18.89
#7	283,551	567,102	84,484	1,539	173,585	30.61
#8	97,015	194,030	21,928	104	44,168	22.76
#10	445,975	891,950	158,529	75,425	543,333	60.92

**Table 10:** Saved data.

i	Contour #1. (Outer Contour)					Contour #2. (Inner Contour)				
	$R_{i}$		$C_{i}$			$R_{i}$		$C_{i}$		
	*x*	*y*	*x*	*y*	type	*x*	*y*	*x*	*y*	type
1	3.5	1.5	9.5	9.5	NE-SW	5.5	9.5	5.5	9.5	NW-SE
2	5.5	3.5	5.5	9.5	NW-SE	9.5	5.5	9.5	9.5	NE-SW
3	9.5	1.5								
4	11.5	3.5								
5	13.5	7.5								
6	11.5	9.5								
7	9.5	11.5								
8	5.5	9.5								
9	3.5	7.5								
10	1.5	3.5								

**Table 11:** Increases of pixels traced by the bidirectional scan from the one-directional scan.

	Total Number	Proposed		ISBF		MSBF		SBF		MNT		RSA	
		Number	%	Number	%	Number	%	Number	%	Number	%	Number	%
#1	81,189	1	0.001	1	0.001	1	0.001	1	0.001	0	0.000	0	0.000
#2	50,825	0	0.000	0	0.000	0	0.000	0	0.000	0	0.000	0	0.000
#3	152,489	2	0.001	2	0.001	3	0.002	3	0.002	1	0.001	1	0.001
#4	312,812	0	0.000	0	0.000	0	0.000	0	0.000	0	0.000	0	0.000
#5	157,377	3	0.002	3	0.002	0	0.000	0	0.000	0	0.000	0	0.000
#6	98,579	7	0.007	7	0.007	8	0.008	8	0.008	1	0.001	1	0.001
#7	283,717	120	0.042	120	0.042	67	0.024	67	0.024	92	0.032	92	0.032
#8	97,031	12	0.012	12	0.012	15	0.015	15	0.015	1	0.001	1	0.001
#9	453,721	7075	1.559	7076	1.560	433	0.095	444	0.098	3414	0.752	3414	0.752
Total	1,687,740	7220	0.428	7221	0.428	527	0.031	538	0.032	3509	0.208	3509	0.208

**Table 12:** Computational time traced bidirectionally.

Image	Proposed		ISBF		MSBF		SBF		MNT		RSA	
	Processing	Ratio	Processing	Ratio	Processing	Ratio	Processing	Ratio	Processing	Ratio	Processing	Ratio
	Time		Time		Time		Time		Time		Time	
#1	0.064	2.1	0.067	2.2	0.074	2.2	0.064	2.0	0.067	2.1	0.069	1.8
#2	0.061	2.8	0.063	2.2	0.061	2.1	0.056	2.4	0.061	2.6	0.067	2.4
#3	0.072	1.9	0.083	2.1	0.084	1.8	0.077	2.0	0.077	1.9	0.083	1.8
#4	0.11	1.6	0.116	1.5	0.131	1.4	0.109	1.6	0.111	1.6	0.135	1.5
#5	0.078	1.8	0.084	1.9	0.089	1.8	0.078	1.8	0.078	1.9	0.089	1.8
#6	0.065	2.2	0.073	2.1	0.075	2.0	0.064	1.9	0.07	2.2	0.072	2.1
#7	0.108	1.7	0.111	1.5	0.134	1.4	0.11	1.6	0.113	1.7	0.125	1.4
#8	0.069	2.2	0.07	2.0	0.074	1.9	0.067	2.2	0.067	2.2	0.075	2.3
#9	0.128	1.5	0.139	1.5	0.172	1.3	0.127	1.4	0.13	1.5	0.147	1.3
Total	0.755	1.8	0.806	1.8	0.894	1.6	0.752	1.8	0.774	1.8	0.862	1.7
